# Lipopolysaccharide-induced interleukin-6 production is controlled by glycogen synthase kinase-3 and STAT3 in the brain

**DOI:** 10.1186/1742-2094-6-9

**Published:** 2009-03-11

**Authors:** Eléonore Beurel, Richard S Jope

**Affiliations:** 1 Department of Psychiatry and Behavioral Neurobiology, University of Alabama at Birmingham, Birmingham, AL 35294-0017, USA

## Abstract

**Background:**

Septic shock is a prevalent condition that, when not lethal, often causes disturbances in cognition, mood, and behavior, particularly due to central actions of the inflammatory cytokine interleukin-6 (IL-6). To identify potential targets to control brain IL-6, we tested if IL-6 produced by glia is regulated by signal transducer and activator of transcription-3 (STAT3) and glycogen synthase kinase-3 (GSK3).

**Methods:**

Lipopolysaccharide (LPS) was used to induce inflammatory responses in mice or cultured primary glia. IL-6 was measured by ELISA and other inflammatory molecules were measured using an array.

**Results:**

Mouse brain IL-6 levels increased after central, as well as peripheral, LPS administration, consistent with glia producing a portion of brain IL-6. STAT3 in the brain was activated after peripheral or central LPS administration, and in LPS-stimulated cultured primary glia. Inhibition of STAT3 expression, function, or activation reduced by ~80% IL-6 production by primary glia, demonstrating the dependence on active STAT3. GSK3 promotes STAT3 activation, and array analysis of inflammatory molecules produced by LPS-stimulated primary glia demonstrated that IL-6 was the cytokine most diminished (>90%) by GSK3 inhibition. Inhibition of GSK3, and knockdown of GSK3β, not GSK3α, greatly inhibited IL-6 production by LPS-stimulated primary glia. Conversely, expression of active STAT3 and active GSK3 promoted IL-6 production. In vivo inhibition of GSK3 reduced serum and brain IL-6 levels, brain STAT3 activation, and GFAP upregulation following LPS administration.

**Conclusion:**

STAT3 and GSK3 cooperatively promote neuroinflammation, providing novel targets for anti-inflammatory intervention.

## Background

The inflammatory system is hyperactivated during sepsis, a potentially lethal condition induced by bacterial infection that affects nearly 1 million people in the United States every year [1]. Inflammation is controlled by a balance of activating and inhibitory signals delivered intracellularly by transmembrane receptors that recognize components of invasive bacteria [2]. Sepsis ensues due to hyperactivation of the innate immune system that causes a massive production of proinflammatory cytokines and chemokines that cause vascular leakage and septic shock, impairing the function of vital organs [1]. Encephalopathy is a common feature in sepsis, often occurring before failure of other organs such as kidney, liver and lung. Surviving individuals often suffer deleterious consequences of sepsis, such as cognitive deficits and other signs of long-term impairments in the central nervous system (CNS) [3,4].

Interleukin-6 (IL-6) is considered one of the major markers of lethal sepsis [5], for example as demonstrated in studies using IL-6 knockout mice [6] but is not a target for treatment because in short-term mortality studies anti-IL-6 strategies were unsuccessful [7]. However, increased brain IL-6 has been associated with severe cognitive impairments [8-10] and likely contributes to the cognitive and neuroanatomical long-term consequences of sepsis, such as persistent behavioral deficits and neuronal loss [11]. These findings indicate that strategies to reduce IL-6 production may be particularly valuable for protecting the CNS from damage caused by sepsis.

Recently, glycogen synthase kinase-3 (GSK3) was identified as a crucial regulator of innate inflammatory processes [12,13]. GSK3 is a constitutively active Ser/Thr kinase consisting of two isoforms, GSK3α and GSK3β. GSK3 was found to strongly promote Toll-like receptor (TLR)-induced production of several pro-inflammatory cytokines in monocytes, and GSK3 inhibition rescued 60–70% of mice from an otherwise lethal septic shock [12]. Subsequently, inhibition of GSK3 was shown to protect rodents from several peripheral inflammatory conditions (reviewed in [14]). Members of the signal transducer and activator of transcription (STAT) family of transcription factors have central roles in inflammatory reactions, and STAT3 was considered anti-inflammatory [15], mediating SOCS-3 or IL-10 signals, and endothelial STAT3 contributes to anti-inflammatory responses to LPS [16]. Although STAT3 is activated in numerous neuropathological conditions such as autoimmune encephalomyelitis [17] and ischemia [18] and has been implicated in reactive astrogliosis [19], the inflammatory role of STAT3 in the brain is poorly understood. We report here that in contrast to its systemic role, STAT3 has proinflammatory properties in the context of septic shock-induced neuroinflammation. This occurs in cooperation with proinflammatory GSK3, which is known to participate to the activation of STAT3 [20], as inhibition of STAT3 or GSK3 greatly reduced IL-6 production by stimulated glia. These results identify STAT3 and GSK3 as potential targets to control brain IL-6 production and neuroinflammation.

## Methods

### Materials

Protein-free *E. coli *(K235) LPS was prepared as described [12]. Sources of substances used and solvents were as follows: IFNγ (R&D Systems), SB216763 (in DMSO; Tocris), kenpaullone, indirubin-3'-monoxime, BIO (6-bromoindirubin-3'-oxime), GSK3 inhibitor II, AG490 (each in DMSO; Calbiochem), LiCl (in water; Sigma), and JSI-124 (cucurbitacin, in DMSO; National Cancer Institute Developmental Therapeutic Program). Mouse IL-1β, TNFα, IL-6, IL-10 and IFNγ were measured with ELISA kits according to the manufacturer's instructions (eBioscience). The concentrations of 62 inflammatory proteins (listed in Additional files 1 and 2) were measured with inflammatory molecule antibody arrays according to the manufacturer's instructions (Raybiotech).

### Mice

Male C57Bl/6 mice (8–10 weeks) were housed in a light and temperature controlled room, Mice were injected intraperitoneally (ip) with *E. col*i K235 LPS (10 μg/g in 200 μL saline) followed by measurements of cytokines by ELISA in the serum and brain regions. In some mice, lithium was administered in pelleted food containing 0.2% lithium carbonate (Harlan-Teklad) for 3 weeks before LPS treatment, which resulted in serum lithium concentrations of 0.53 ± 0.03 mM (n = 4). For central administration using ketamine/xylazine (100 mg/kg, 10 mg/kg) anesthesia, LPS (10 μg/1 μL) was slowly infused stereotaxically into each lateral ventricle (icv; relative to bregma 0.4 mm posterior, ± 1 mm lateral, 2.2 mm depth), mice were sacrificed 2, 4, or 8 hr after treatment, and tissue from the hippocampus adjacent to the ventricle (ventricular infusion zone) and from the distant occipital cortex was analyzed for cytokines by ELISA. For immunoblotting, brain regions were homogenized in ice-cold lysis buffer containing 20 mM Tris-HCl, pH 7.4, 150 mM NaCl, 2 mM EDTA, 1% Triton X-100, 10% glycerol, 1 μg/mL of leupeptin, aprotinin, and pepstatinA, 1 mM orthovanadate, 50 mM NaF, 0.1 μM okadaic acid, 1 mM PMSF. The lysates were centrifuged at 14 000 rpm for 10 min to remove insoluble debris. Protein concentrations in the supernatants were determined in duplicate using the Bradford protein assay. Mice were housed and treated in accordance with National Institutes of Health and the University of Alabama at Birmingham Institutional Animal Care and Use Committee guidelines.

### Cell culture, immunoblotting, and cell viability assay

For murine primary neurons, the hippocampus from 1 day old C57Bl/6 mice was isolated and incubated in 0.1% trypsin (Invitrogen) and the cells mechanically dissociated using a fire-polished pipette. Cells were plated in DMEM/F12 medium supplemented with 10% FBS, 0.3% glucose, 2 mM glutamine, 10 U/mL penicillin and 10 μg/mL streptomycin in poly-D-lysine-coated six-well plates. Twelve hours after plating, the medium was replaced with Neurobasal medium supplemented with B27 and 0.5 mM glutamine (Invitrogen) to promote neuronal survival and to inhibit the growth of non-neuronal cells. Neurons were used for experiments after seven days in culture. Primary glia were prepared from the cerebral cortex of 1 day old C57Bl/6 mice as described [21], cultured in DMEM/F12 medium supplemented with 10% FBS, 0.3% glucose, 2 mM L-glutamine, 10 U/mL penicillin and 10 μg/mL streptomycin. For separation of astrocytes and microglia, after 10 days of culture the cells were shaken (30 hr; 250 rpm), resulting in >99% pure astrocytes as determined by immunostaining with the astrocyte marker glial fibrillary acidic protein (GFAP). After the first hour of shaking, the medium containing microglia cells was collected and microglia were cultured in the same medium as astrocytes. For expression experiments, cells were rinsed twice with DMEM/F12 medium without supplements, infected with 50 moi of the designated adenovirus for 30 min, supplemented medium was added for incubation for 36–48 h, and infected cultures were examined for adequate infection efficiency (80%) as assessed by GFP fluorescence after infection with GFP adenovirus. For knockdown experiments, cells were transfected using liposome-mediated transfection reagent LipofectAMINE RNAiMAX (Invitrogen) with 50 nM siRNA according to the manufacturer's instructions with STAT3 prevalidated siRNA [22], silencer negative control (Ambion), or GSK3α and GSK3β siRNA (Smart pool; Dharmacon). For experimental treatments, cells were pretreated with the indicated inhibitors for 30 min followed by treatment with 100 ng/mL LPS, 1 ng/mL IFNγ, or both, for the indicated times. Following treatments, cells were washed twice with phosphate-buffered saline (PBS) and were lysed with lysis buffer (20 mM Tris-HCl, pH 7.4, 150 mM NaCl, 2 mM EDTA, 1% Triton X-100, 10% glycerol, 1 μg/mL of leupeptin, aprotinin, and pepstatinA, 1 mM orthovanadate, 50 mM NaF, 0.1 μM okadaic acid, 1 mM phenylmethanesulfonyl fluoride). The lysates were centrifuged at 14 000 rpm for 10 min. Protein concentrations were determined in duplicate using the Bradford protein assay. Immunoblotting was carried out as described before [20] using antibodies to phospho-Tyr705-STAT3, total STAT3 (Cell Signaling Technology), GFAP, GSK3α/β (Millipore), and β-actin (Sigma). Immunoblots were developed using horseradish peroxidase-conjugated goat anti-mouse, or goat anti-rabbit IgG, followed by detection with enhanced chemiluminescence, and the protein bands were quantitated with a densitometer. Cell viability was assessed by a colorimetric 3-(4,5-dimethylthiazol-2-yl)-2,5-diphenyltetrazolium bromide (MTT) assay. A multi-well scanner was used to measure the absorbance at 570–630 nm dual wavelengths. Statistical significance between groups was evaluated by Student's *t*-test.

## Results

### IL-6 robustly accumulates in the brain after sepsis

**Figure 1:**
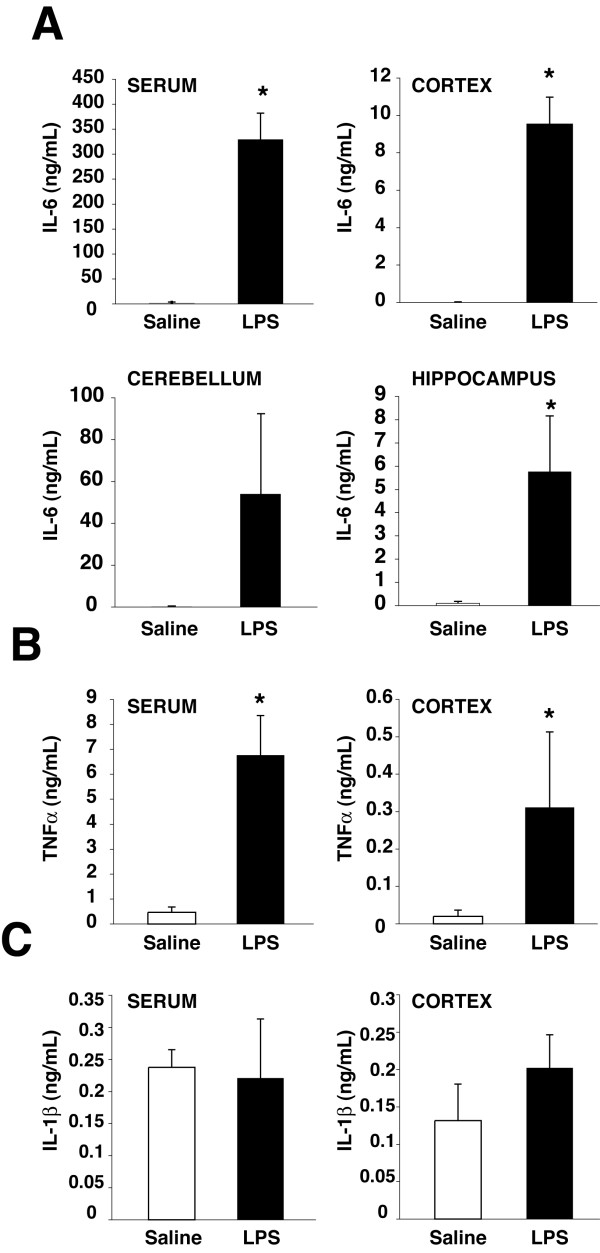
**Levels of cytokines in plasma and the brain parenchyma in response to sepsis**. Serum and brain region levels of A, IL-6, B, TNFα, and C, IL-1β were measured 4 h after ip injection of 10 μg/g LPS or saline to evaluate the early outcomes of acute sepsis. Means ± SEM; n = 3;* p < 0.05.

**Figure 2:**
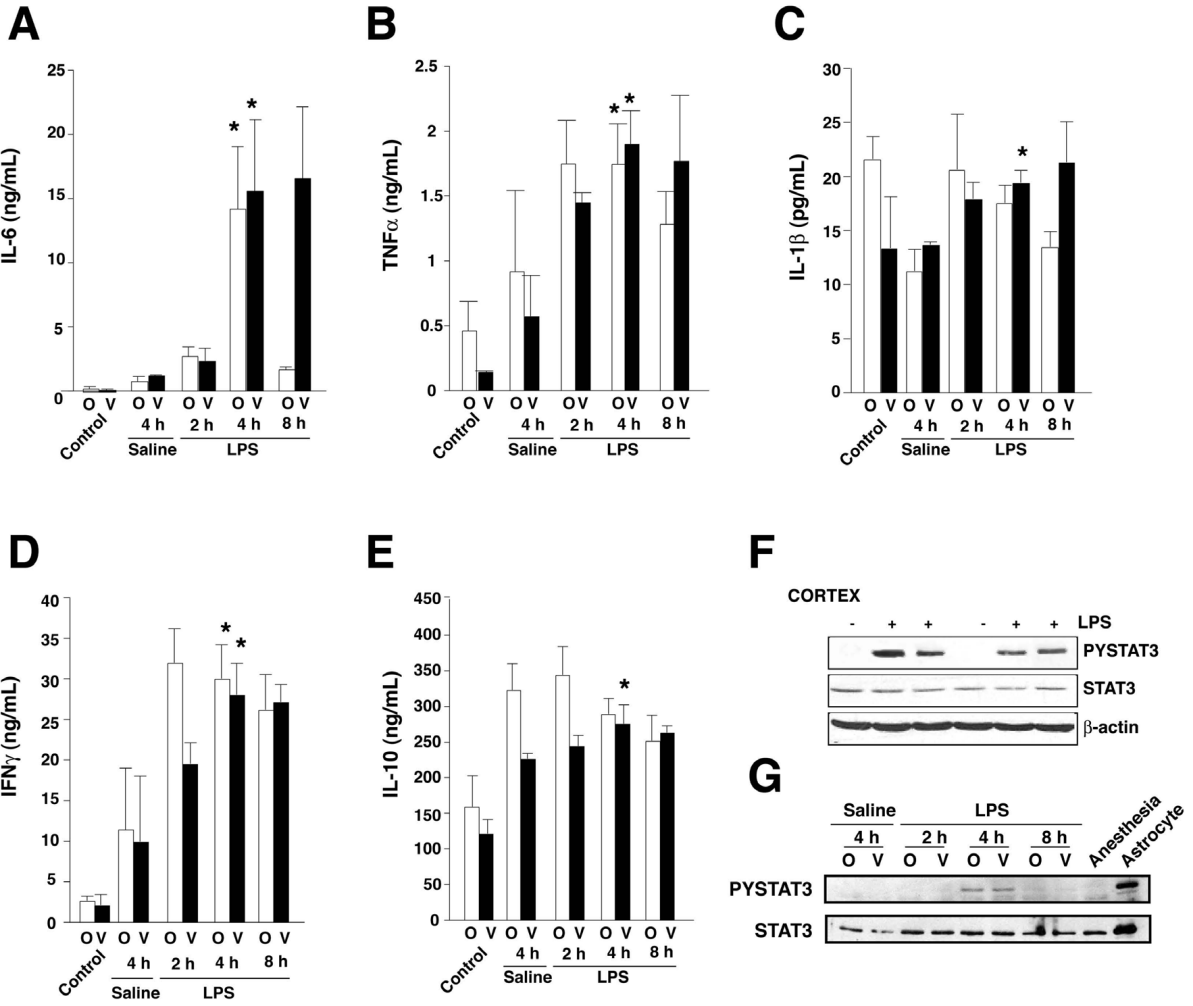
**Brain cytokines and STAT3 activation**. A, IL-6, B, TNFα, C, IL-1β, D, IFNγ, and E, IL-10, were measured in homogenates (100 μg) of the ventricular infusion zone (V) or the distant occipital cortex (O), 2, 4 or 8 h after LPS (10 μg/1 μL; icv) or 4 h after saline (icv) injection. Control mice were not subjected to surgery. Means ± SEM.; n = 4–5 mice/group, *p < 0.05 4 hr treatments compared to mice injected with saline. Activation of STAT3 was measured by immunoblotting phospho-Tyr705-STAT3 and total STAT3, F, in cerebral cortex 4 h after ip injection of LPS or, G, in the ventricular infusion zone (V) or occipital cortex (O) 2, 4 or 8 h after icv infusion of LPS or 4 h after saline and compared to mice subjected to anesthesia only. STAT3 immunoblotted from cultured astrocytes is shown as a positive control.

IL-6 in the cerebral cortex, cerebellum, and hippocampus was markedly increased 4 hr after peripheral LPS administration, which reflected the increased serum IL-6 level, whereas LPS administration slightly increased cortical and serum levels of TNFα, but not of IL-1β, at this time (Figure 1A–C). IL-6 present in brain regions was likely partly produced in the brain because after icv administration of LPS, IL-6 accumulated both in the ventricular infusion zone and in the distant occipital cortex, indicating centrally-induced spreading inflammation (Figure 2A). Central LPS infusion also moderately increased brain levels of TNFα and IFNγ in both brain regions but with a persistence in the ventricular infusion zone, while IL-1β and the anti-inflammatory cytokine IL-10 levels slightly increased at late time points in the ventricular infusion zone only (Figure 2B–E). Administration of saline vehicle had more modest effects than LPS on the levels of cytokines in the brain. Thus, in response to sepsis, IL-6 robustly accumulates in the brain and is at least partly produced by the central inflammatory system, although some contribution from the periphery cannot be ruled out.

**Figure 3:**
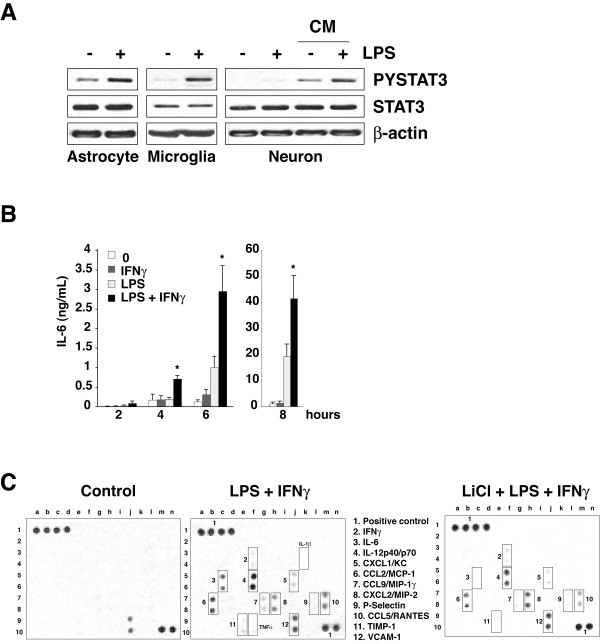
**STAT3 activation and cytokine production in cultured cells**. A, Phospho-Tyr705-STAT3 and total STAT3 were immunoblotted after treatment with LPS (100 ng/mL) for 6 h of primary cultures of astrocytes, microglia, or neurons. Some of the neuronal cultures also were treated for 6 h with conditioned media (CM) from LPS-treated (6 h) astrocytes. B, Primary enriched astrocytes were treated with vehicle (0), or with 100 ng/mL LPS, 1 ng/mL IFNγ, or both, for 2, 4, 6, or 8 h, followed by measurements of IL-6. Means ± SEM; n = 5; *p < 0.05 compared to LPS alone. C, Primary enriched astrocytes were stimulated with LPS (100 ng/mL) and IFNγ (1 ng/mL), with or without the selective GSK3 inhibitor lithium (LiCl, 20 mM), for 6 h. Culture supernatants were incubated with an array membrane that detects 62 proteins in duplicate. Boxes indicate the location of the detected 13 proteins induced by LPS plus IFNγ. Positive controls are in the upper left and lower right corners (high intensity spots) and negative controls in the upper right corner (no spots).

IL-6 and other cytokines, such as interferons, induce the activation of STAT3 by receptor-induced Janus kinase (JAK)-mediated tyrosine-705 phosphorylation [23]. Phospho-Tyr705-STAT3 in the brain was increased 4 hr after either ip (Figure 2F) or icv LPS administration (Figure 2G), confirming that STAT3 is activated in the brain after sepsis [24,25]. Examined 6 hr after treatment with LPS (100 ng/mL), STAT3 was activated in primary astrocytes and microglia (Figure 3A). Treatment with LPS alone did not stimulate STAT3 in primary neurons, but neuronal STAT3 was activated after treatment with conditioned medium from treated astrocytes, indicating that a factor released from glia, such as IL-6, can stimulate neuronal STAT3. Thus, STAT3 can be activated in all three types of cells during in vivo inflammation.

The production of IL-6 was also evaluated in primary glia after stimulation with LPS or with IFNγ co-administration with LPS, which amplifies LPS-induced cytokine production. There was a time-dependent increase in IL-6 production in response to LPS (100 ng/mL) that was amplified by co-stimulation with 1 ng/mL IFNγ (Figure 3B). Inflammatory molecule array analysis showed a large increase in the production of IL-6 by primary glia in response to stimulation with LPS plus IFNγ (Figure 3C). Several other products implicated in the pathogenesis of sepsis were also induced by this treatment, including IL-12p40, CCL5/RANTES, CXCL1/KC, CCL9/MIP-1γ, CXCL2/MIP-2, P-Selectin, TIMP-1 and CCL2/MCP-1 (Figure 3C).

### STAT3 participates in IL-6 production

**Figure 4:**
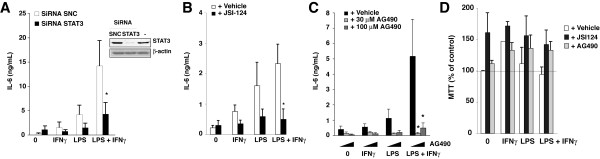
**STAT3 contributes to the LPS-induced IL-6 production in primary enriched astrocytes**. Primary enriched astrocytes were treated with 100 ng/mL LPS, 1 ng/mL IFNγ, or both, for 6 h, followed by measurements of IL-6. A, Inhibition of IL-6 production after 36 h siRNA-mediated knockdown of STAT3. The knockdown efficiency was ensured by immunoblotting for STAT3 (*inset*). (n = 6,*p < 0.05 compared to LPS plus IFNγ with control siRNA SNC). B, Inhibition of LPS plus IFNγ (6 h) stimulated IL-6 production by inhibition of STAT3 with 10 μM JSI-124 (n = 8, *p < 0.05 compared to LPS plus IFNγ with DMSO). C, Inhibition of LPS plus IFNγ (6 h) stimulated IL-6 production by treatment with 30 (middle bars, light shade) or 100 μM AG490 (right bars, medium shading) (n = 5, *p < 0.05 compared to LPS plus IFNγ with DMSO). Means ± SEM. D, Cell viability: Primary enriched astrocytes were treated with vehicle DMSO (left, white bars), 100 ng/mL LPS, 1 ng/mL IFNγ, or both, and with 10 μM JSI-124 or 100 μM AG490 for 6 h and viability was assessed by a MTT assay. Means ± SEM; n = 3.

To examine the mechanisms regulating the production of IL-6, we tested if STAT3 not only was activated by IL-6 but also participated in a feed-forward loop promoting IL-6 production in primary glia as was recently identified in B lymphocytes [26]. IL-6 production in glia induced by treatment with LPS plus IFNγ was greatly reduced by downregulation of STAT3 expression by siRNA (Figure 4A), by pharmacological inhibition with the STAT3-specific inhibitor JSI-124 (cucurbitacin) [27] (Figure 4B), or with the JAK2 inhibitor AG490 (Figure 4C), neither of which impaired cell viability (Figure 4D). These findings indicate that STAT3 may not only propagate the IL-6 signal after sepsis but also contributes to inflammation-induced IL-6 production in glia.

### IL-6 production is strongly dependent on GSK3

**Figure 5:**
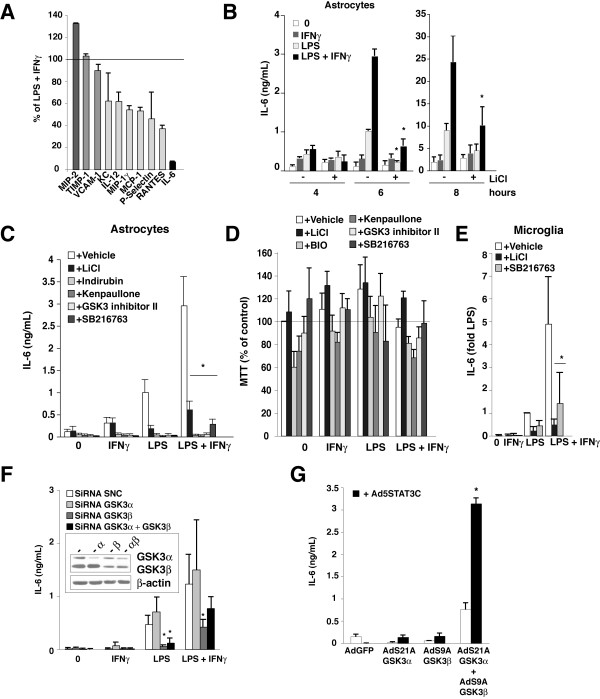
**GSK3 promotes IL-6 production**. A, Quantitation of the effects of lithium treatment on inflammatory molecules produced by primary enriched astrocytes stimulated with LPS (100 ng/mL) and IFNγ (1 ng/mL) for 6 h and analyzed by cytokine array, as described in Figure 3C. B, Time-dependent production of IL-6 by primary enriched astrocytes treated with vehicle (0), LPS (100 ng/mL), IFNγ (1 ng/mL), or both, in the absence and presence of 20 mM lithium. White bars indicate that no GSK3 inhibitor was added. *p < 0.05 compared to LPS plus IFNγ. C, IL-6 production by primary enriched astrocytes (n = 7) treated with 1 ng/mL IFNγ, 100 ng/mL LPS, or both, without or with 20 mM lithium or 10 μM other GSK3 inhibitors, for 6 h. White bars indicate DMSO vehicle with no GSK3 inhibitor. *p < 0.05 compared to LPS plus IFNγ without any GSK3 inhibitor. D, Cell viability: Primary enriched astrocytes were treated with 100 ng/mL LPS, 1 ng/mL IFNγ, or both, and with 20 mM lithium, 10 μM 6-bromoindirubin-3'-oxime (BIO), 10 μM kenpaullone, 10 μM GSK3 inhibitor II, or 10 μM SB216763 for 6 h and viability was assessed by a MTT assay. White bars indicate DMSO vehicle with no GSK3 inhibitor. Means ± SEM; n = 3. E, IL-6 production by primary microglia (n = 4) treated with 1 ng/mL IFNγ, 100 ng/mL LPS, or both, without or with 20 mM lithium or 10 μM SB216763, for 6 h. White bars indicate that no GSK3 inhibitor was added. *p < 0.05 compared to LPS plus IFNγ without any GSK3 inhibitor. F, IL-6 production by primary enriched astrocytes after 48 h siRNA-mediated knockdown of GSK3α, GSK3β, or both, and stimulation with LPS (100 ng/mL), IFNγ (1 ng/mL), or both, for 6 h (n = 5, *p < 0.05 compared to corresponding control siRNA SNC values). G, Cooperation between GSK3 and STAT3 to induce IL-6 production by primary enriched astrocytes, after 36 h expression of control GFP, S21A-GSK3α, or S9A-GSK3β, with or without expression of STAT3C (n = 3;*p < 0.05). Means ± SEM.

Since STAT3 activation requires active GSK3 [20], systemic IL-6 production is controlled by GSK3 [12], and GSK3 is abundantly expressed in the brain, we tested if GSK3 controls IL-6 production by astrocytes and microglia, first with the selective inhibitor lithium [28] using a cytokine array. Among the 12 molecules detected to be induced by treatment with LPS plus IFNγ in glia, the production of TIMP-1 and VCAM-1 were independent of GSK3, whereas active GSK3 decreased the expression of CXCL2/MIP-2 and increased the expression of CXCL1/KC, IL-12p40, CCL9/MIP-1γ, CCL2/MCP-1, P-Selectin and CCL5/RANTES (Figure 3C and 5A). However, most prominently, active GSK3 promoted IL-6 production by 16-fold, showing that IL-6 production by glia is strongly dependent on GSK3. Confirmation by ELISA showed that inhibition of GSK3 with lithium treatment reduced the time-dependent production of IL-6 induced by LPS and its potentiation by IFNγ in primary astrocytes (Figure 5B). The dependency of IL-6 production in primary astrocytes on GSK3 was confirmed by the strong inhibition exerted by four other structurally diverse GSK3 inhibitors on IL-6 produced in response to LPS or to a combination of LPS plus IFNγ (Figure 5C), which was not due to changes in cell viability (Figure 5D). Inhibition of GSK3 also strongly reduced IL-6 production in primary microglia (Figure 5E). siRNA-mediated knockdown by 75% of GSK3β, but not of GSK3α, greatly reduced the production of IL-6 by primary enriched astrocytes induced by LPS and its potentiation by IFNγ (Figure 5F), indicating that GSK3β predominantly regulates IL-6 production.

### GSK3 and STAT3 are sufficient to stimulate IL-6 production in glia

Since both active STAT3 and active GSK3 were necessary for IL-6 production, the expression of active forms of each was introduced using adenoviruses in primary enriched astrocytes to examine their effects on IL-6 production. These included STAT3C, in which covalent bonds between cysteines establishes the active dimer conformation [29], and constitutively active S9A-GSK3β and S21A-GSK3α, where serines subject to inhibitory phosphorylation were mutated to alanines to prohibit inhibitory serine phosphorylation. Expression of active S21A-GSK3α and S9A-GSK3β together increased IL-6 production and this was increased a further 4.5-fold with co-expression of STAT3C (Figure 5G). This demonstrates that GSK3 and STAT3 were both necessary, and together sufficient, to stimulate IL-6 production in glia cells.

### GSK3 inhibition reduces IL-6 signaling in the brain

**Figure 6:**
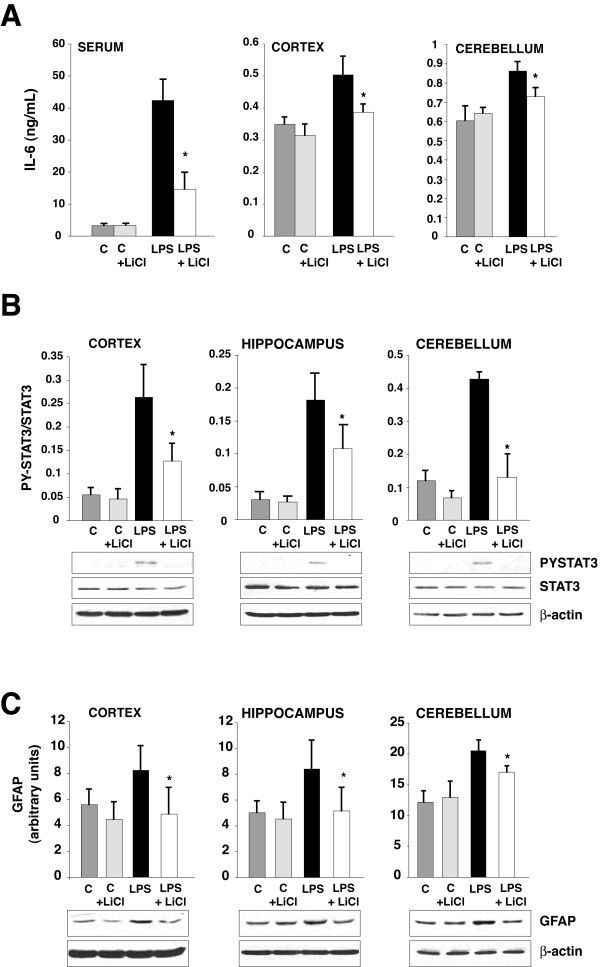
**GSK3 inhibition attenuates IL-6 production and astrogliosis**. Mice were fed for 3 weeks without or with lithium chow. A, Serum and brain region homogenate (100 μg protein) levels of IL-6 were measured 18 h after ip injection of LPS or saline vehicle control (C) to evaluate the relative long-term outcomes of acute sepsis (n = 4–6 mice/group, *p < 0.05, compared to mice injected with LPS without lithium). B, Activation of STAT3 was measured by immunoblotting phospho-Tyr705-STAT3 in brain regions 18 h after ip injection of LPS. Means ± SEM (n = 4–6 mice/group, *p < 0.05 compared to mice injected with LPS without lithium). C, GFAP in brain regions 18 h after LPS (ip). Means ± SEM (n = 4–6 mice/group,*p < 0.05, compared to mice injected with LPS without lithium).

The capacity of GSK3 inhibitors to dampen IL-6 production in glia raised the possibility that GSK3 inhibitors may be beneficial for reducing neuroinflammation. To test this hypothesis, mice were treated with lithium because it is the only GSK3 inhibitor that is well-established to be effective in the CNS after peripheral administration [26]. In mice treated with lithium, the serum IL-6 level 18 hr after LPS administration was 67% lower than in mice not given lithium (Figure 6A). This matches a previous report that the GSK3 inhibitor SB216763 greatly reduced serum IL-6 after LPS challenge [12]. IL-6 levels were elevated in the cerebral cortex and cerebellum (Figure 6A), but not hippocampus (not shown) 18 hr after LPS administration, and lithium pretreatment reduced these increases in IL-6. In mice pretreated with lithium, there was also a significant reduction in the activating tyrosine-phosphorylation of STAT3 following LPS administration in the cerebral cortex, hippocampus, and cerebellum (Figure 6B). Furthermore, inhibition of GSK3 with lithium also reduced the activation of astrocytes, as measured by the astrocyte marker GFAP immunoreactivity, following LPS treatment (Figure 6C). Since STAT3 promotes GFAP expression [30], this indicates that lithium's inhibition of IL-6 production and STAT3 activation reduces expression of STAT3-dependent astrocyte-specific genes such as GFAP, resulting in reduction of astrogliosis induced by LPS.

## Discussion

The inflammatory response mounted in the brain in response to many types of insults is crucial to control and counteract detrimental effects of the insults on neurons. However, neuroinflammation that is severe or chronic can itself damage neurons due to excessive production of cytokines and other inflammatory molecules by astrocytes and microglia [31,32]. Therefore, identification of mechanisms capable of controlling neuroinflammation provide avenues to interrupt the inflammatory process to prevent its deleterious consequences. Here, we identify novel cooperative actions of STAT3 and GSK3 that control IL-6 production by glia during sepsis-induced neuroinflammation. The production of IL-6 by glia was largely blocked by interventions inhibiting the activity of STAT3 or GSK3β, revealing the strong dependence of IL-6 production in glia on these signaling molecules.

Sepsis caused a large increase in IL-6 in the serum, some of which likely accounted for the increased brain levels of IL-6. However, IL-6 also is produced locally in the brain since its level increased after centrally administered LPS and it was produced by LPS-stimulated primary astrocytes and microglia in culture. IL-6 in the brain is apparently a dual-edged sword, having both beneficial effects but also deleterious effects [33], likely depending on the magnitude and duration of its stimulated production. IL-6 may have acute neuroprotective and regenerative effects on neurons [34], which have been reported to depend on its ability to inhibit the synthesis of TNF, induce nerve growth factor, and promote neuronal differentiation and survival [35]. However, chronic or high increases in IL-6 levels in the brain lead to neuronal and cognitive impairments [8-11]. The actions of IL-6 are partly mediated by its effects on glia, but it also is not clear if the effects of IL-6 on astrocytes and microglia are beneficial or detrimental. IL-6 is considered an important inducer of astrogliosis, promoting reactive responses regulating the activation state of astrocytes [36,37], but chronic or excessive activation of glia is often associated with neuronal loss and may contribute to many widespread neurodegenerative diseases, such as Alzheimer's disease and multiple sclerosis [38].

Our findings indicate that inhibition of STAT3 and GSK3 provide mechanisms to intervene to reduce IL-6 production. In glia, IL-6 production was highly dependent on the transcription factor STAT3, suggesting that by activating STAT3 IL-6 promoted its own production, as well as activating astrocytes as indicated by increases in the astrocyte reactive marker GFAP. This amplification loop was recently identified in B cells, as B cell-lymphoma with high expression of STAT3 exhibited elevated production of IL-6 [26]. Our study expands this observation by showing this mechanism of a feed-forward loop is activated in the brain after sepsis. Thus, this may provide a target to specifically dampen the effects of IL-6 on glia after sepsis. GSK3 also was required for IL-6 production, as inhibitors of GSK3 greatly attenuated IL-6 production. Although other cytokines and chemokines also were regulated by GSK3 inhibitors, IL-6 displayed the greatest dependence on GSK3. These findings likely signify cooperative actions of STAT3 and GSK3 in promoting IL-6 production since STAT3 activation is dependent on GSK3 [20]. Thus, GSK3 is capable of promoting IL-6 production by contributing to the activation of both STAT3 and NF-κB [39].

## Conclusion

The identification of GSK3 as a regulator of IL-6 production in the brain expands the already well-known role of GSK3 as an integrator of many signaling pathways involved in inflammatory signaling [14] and indicates that inhibition of GSK3 may provide a new therapeutic strategy to counteract the detrimental consequences of sepsis in the brain. Furthermore, in contrast to interventions targeting individual transcription factors, inhibiting GSK3 enables simultaneous regulation of multiple transcription factors involved in inflammatory signaling, a therapeutic potential strengthened by the rapidly growing armament of GSK3 inhibitors that includes lithium, a drug already used therapeutically in human patients. These agents may contribute to the regulation of inflammation, which continues to be a pressing problem because of the widespread association of excessive inflammation with many diseases, and particularly neuroinflammation, which contributes to a broad range of neurodegenerative diseases.

## Competing interests

The authors declare that they have no competing interests.

## Authors' contributions

EB carried out the experimental work and both authors contributed to the design, analysis of the data, and manuscript preparation.

## Supplementary Material

Additional File 1 **Table 1: Proteins measured with the cytokine antibody array**. Substances regulated by GSK3 inhibition are marked in gray. Click here for file

Additional File 2 **Table 2: Abbreviations of the proteins measured with the cytokine antibody array**. Click here for file
