# Supplementary figures and images for: Lipopolysaccharide-induced interleukin-6 production is controlled by glycogen synthase kinase-3 and STAT3 in the brain
Source: J Neuroinflammation. 2009 Mar 11;6:9. doi: 10.1186/1742-2094-6-9 (PMC2660311; doi:10.1186/1742-2094-6-9)

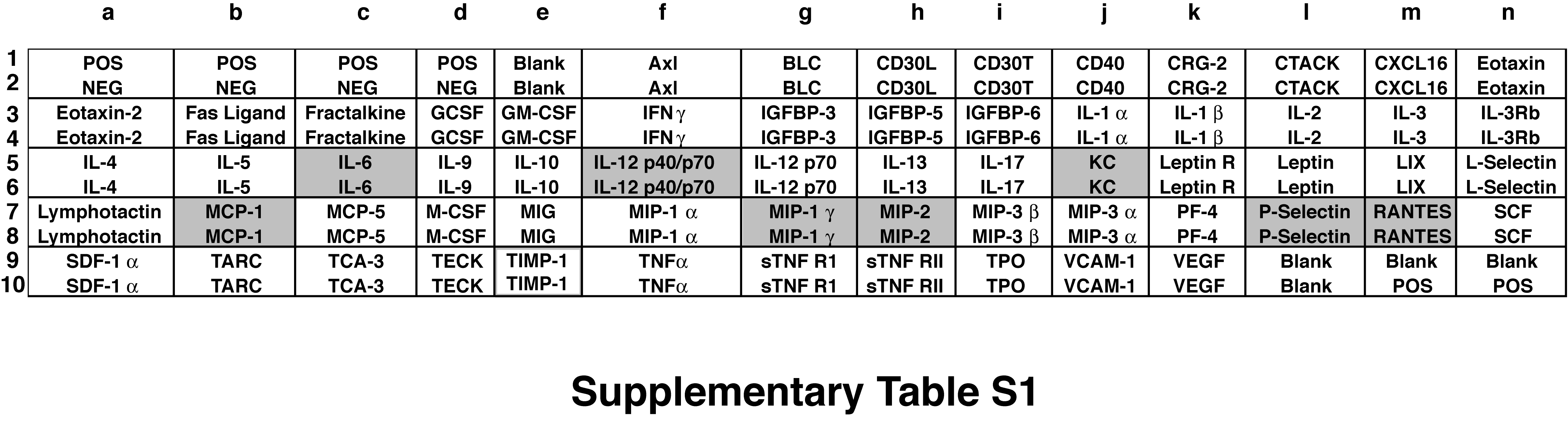

Supplement: Additional File 1 — Table 1: Proteins measured with the cytokine antibody array. Substances regulated by GSK3 inhibition are marked in gray. [file 1742-2094-6-9-S1.tiff]
