# Supplementary material for: Lipopolysaccharide-induced interleukin-6 production is controlled by glycogen synthase kinase-3 and STAT3 in the brain
Source: J Neuroinflammation. 2009 Mar 11;6:9. doi: 10.1186/1742-2094-6-9 (PMC2660311; doi:10.1186/1742-2094-6-9)
Supplement: Additional File 2 — Table 2: Abbreviations of the proteins measured with the cytokine antibody array. [file 1742-2094-6-9-S2.pdf]

Supplementary Table S2 :

Protein Name	*Official Name provided from HUGO Gene Nomenclature Commitee (HGNC)*
**AXL**	AXL receptor tyrosine kinase
**CCL1/TCA-3**	Chemokine (C-C motif) ligand 1
**CCL11/Eotaxin**	Chemokine (C-C motif) ligand 11
**CCL12/MCP-5**	Chemokine (C-C motif) ligand 12
**CCL17/TARC**	Chemokine (C-C motif) ligand 17
**CCL19/MIP-3β**	Chemokine (C-C motif) ligand 19
**CCL2/MCP-1**	Chemokine (C-C motif) ligand 2
**CCL20/MIP-3α**	Chemokine (C-C motif) ligand 20
**CCL24/ Eotaxin-2**	Chemokine (C-C motif) ligand 24
**CCL25/TECK**	Chemokine (C-C motif) ligand 25
**CCL27/CTACK**	Chemokine (C-C motif) ligand 27 (Cutaneous T-Cell attracting chemokine)
**CCL3/MIP-1α**	Chemokine (C-C motif) ligand 3
**CCL5/RANTES**	Chemokine (C-C motif) ligand 5
**CCL9/MIP-1γ**	Chemokine (C-C motif) ligand 9
**CD30**	Cluster of differentiation 30
**CD40**	Cluster of differentiation 40
**CX3CL1/Fractalkine**	Chemokine (C-X3-C motif) ligand 1
**CXCL1/KC**	Chemokine (C-X-C motif) ligand 1
**CXCL10/CRG-2**	Chemokine (C-X-C motif) ligand 10
**CXCL12/SDF-1α**	Chemokine (C-X-C motif) ligand 12 (stromal cell-derived factor 1)
**CXCL13/BLC**	Chemokine (C-X-C motif) ligand 13 (B-cell chemoattractant)
**CXCL-16**	Chemokine (C-X-C motif) ligand 16
**CXCL2/MIP-2**	Chemokine (C-X-C motif) ligand 2
**CXCL4/PF-4**	Chemokine (C-X-C motif) ligand 4 (Platelet factor 4)
**CXCL5/LIX**	Chemokine (C-X-C motif) ligand 5 (LPS induced C-X-C chemokine)
**CXCL9/MIG**	Chemokine (C-X-C motif) ligand 9
**Fas Ligand**	Fas ligand
**G-CSF**	Colony stimulating factor 3 (granulocyte)
**GM-CSF**	Colony stimulating factor 2 (granulocyte-macrophage)
**IFNγ**	Interferon, gamma
**IGFBP-3**	Insulin-like growth factor binding protein 3
**IGFBP-5**	Insulin-like growth factor binding protein
**IGFBP-6**	Insulin-like growth factor binding protein 6
**IL-10**	Interleukin-10
**IL-12p40/p70**	Interleukin-12B (natural killer cell stimulatory factor 2, p40)
**IL-12p70**	Interleukin-12A (natural killer cell stimulatory factor 1, p35)
**IL-13**	Interleukin-13
**IL-17**	Interleukin-17
**IL-1α**	Interleukin-1, alpha
**IL-1β**	Interleukin-1, beta
**IL-2**	Interleukin-2
**IL-3**	Interleukin-3
**IL-3Rb**	Interleukin-3 receptor
**IL-4**	Interleukin-4
**IL-5**	Interleukin-5
**IL-6**	Interleukin-6

**IL-9**	Interleukin-9
**Leptin**	Leptin
**LeptinR**	Leptin receptor
**L-Selectin**	Leucocyte Selectin (Lymphocyte adhesion molecule 1)
**M-CSF**	Colony stimulating factor 1 (macrophage)
**P-Selectin**	Platelet selectin
**SCF**	KIT ligand
**sTNFRI**	Soluble TNF receptor I
**sTNFRII**	Soluble TNF receptor II
**TIMP-1**	TIMP metallopeptidase inhibitor 1
**TNFα**	Tumor necrosis factor (TNF superfamily, member 2)
**TPO**	Trombopoietin (megakaryocyte growth and development factor)
**VCAM-1**	Vascular cell adhesion molecule 1
**VEGF**	Vascular endothelial growth factor
**XCL1/Lymphotactin**	Chemokine (C motif) ligand 1
